# “Let’s Talk About It”: The Moderating Role of Self-Disclosure on Complicated Grief over Time among Suicide Survivors

**DOI:** 10.3390/ijerph16193740

**Published:** 2019-10-04

**Authors:** Yossi Levi-Belz, Lilac Lev-Ari

**Affiliations:** 1 Department of Behavioral Sciences, Ruppin Academic Center, Emek Hefer 40250, Israel; ldlevari@ruppin.ac.il; 2 The Lior Tsfaty Center for Suicide and Mental Pain Studies, Ruppin Academic Center, Emek Hefer 40250, Israel

**Keywords:** suicide survivors, complicated grief, self-disclosure, longitudinal

## Abstract

Suicide often imparts highly stressful ramifications to those left behind. Previous research on suicide survivors (SUSs) has demonstrated their being at high risk for developing anxiety and depression, including pathological complicated grief (CG). Self-disclosure (S-D)––the tendency to share one’s personal feelings––has been found to be an important component of dealing with grief. In this study, we examined the effect of S-D on CG in an 18-month longitudinal design following one hundred fifty-six SUSs. We found that SUSs suffering from pathological CG at Time 1 (T1) were lower in S-D at T1 and T2 and higher in depression at T2. We also found that SUSs with lower S-D at T1 had higher CG at T2. Using a structural equation model, we found that S-D at T1 contributed significantly (and negatively) to CG at T1, above and beyond the natural fading of CG over time. Our findings emphasize that while CG is highly prevalent among SUSs, S-D has a beneficial effect which can serve as a protective factor against CG for this group. Implications regarding possible interventions with SUSs were discussed.

## 1. Introduction

The suicide phenomenon is acknowledged to extend well beyond the person who died by suicide. Approximately one million people worldwide die by suicide annually. Current estimates indicate that each incident of suicide results in 135 acquaintances being affected [1] and 25 in the category of suicide survivors (SUSs)––those individuals who were profoundly impacted and bereaved [2]. These numbers highlight the position that suicide affects a large number of people who may need professional help or support following the suicide event [3]. To help identify those needing these services, research efforts should focus on ascertaining facilitators and moderators that could ease the bereavement processes for SUSs.

Edwin Shneidman already stressed that suicide is the end of mental pain and agony for the individual who died by suicide, but also the beginning of a life full of pain and hardship for those left behind [4]. Following this, research has introduced the possibility of the multidimensionality of general grief reactions, which may involve erroneous emotions, health problems, cognitive impairment, and impaired role functioning [5]. However, compared with other bereaved populations, SUSs have been found to have severe deleterious health outcomes, among them depression and anxiety, along with suicidal ideation and attempts [3,6]. One of the most difficult experiences of the bereaved in general and of SUSs specifically is the complicated grief (CG) experience. CG has been defined as prolonged, unresolved, or traumatic grief. CG is characterized by acute grief that remains intense and enduring and does not transition into integrated grief [7], but instead, engenders distress and interference with functioning [8]. The CG experience may include feelings of longing and yearning that do not substantially decrease with time and may experience impairment in daily struggling in the absence of the deceased [7]. Symptoms include intense and recurrent yearning, longing and emotional pain, frequent preoccupying thoughts and memories of the deceased person, inability to accept the loss, mixed with avoidance of reminders of the loss. Individuals with high levels of CG often have substantial deficiencies in their daily, occupational, and social functioning [9]. Furthermore, they have increased rates of psychiatric comorbidity, including health problems, sleep disturbance, substance abuse, higher probability for major depression diagnosis [10], as well as suicidal thinking and behavior [11]. Overall, untreated CG results in anguish, impairment, and poor psychological outcomes that persist in the absence of treatment.

Some reports have suggested that as many as 10% to 20% of bereaved individuals develop CG [12,13,14]. However, SUSs are at higher risk of developing CG [15,16,17], with some studies reporting that almost half of the suicide-bereaved participants endure high levels of CG [18]. This prominence of CG can be explained by its unique type of bereavement, as suicide bereavement is typically accompanied by anger, stigma, guilt, and shame, associated with suicide bereavement [7,19]. Importantly, SUSs with CG are twice as likely to develop recurrent and current depression, compared with other bereaved individuals [17] and almost 10 times more likely to report suicidal ideation following their loved one’s suicide [11]. Thus, it is important to understand the personal and interpersonal moderators that may serve as protective factors against this devastating experience.

Several factors may serve as predictors of complicated grief. In their systematic review, Lobb et al. [20] found that previous loss, a previous psychiatric history, exposure to trauma, and the closeness of the relationship to the deceased are significant predictors of CG levels. Factors of attachment style and related interpersonal traits, such as self-disclosure, also appear to play a critical role in CG among bereaved individuals [20]. However, all of these studies adopted a cross-sectional methodology, thus, precluding any causal. In the current study, we focused on the role of self-disclosure as a moderator of CG among SUSs in a longitudinal design.

### 1.1. Self-Disclosure

*Self-disclosure* (S-D) refers to the process by which persons let themselves be known by others [21]. Numerous studies have demonstrated S-D as a prerequisite for healthy adjustment [22,23]. People who could talk about the death event showed significantly fewer grief difficulties and exhibited a lessening of mental health disturbance [24]. In another interesting study by these authors, Oexle, Feigelman, and Sheehan [25] found that among suicide-loss survivors, those who felt the need to keep their suicide loss secret also exhibited increased grief difficulties and even suicidality. 

SUSs are often characterized by feelings of shame and guilt [26]; therefore, disclosing intimate feelings and thoughts, with significant others might facilitate emotional processing [27]. These emotional processes, in turn, could potentially avert adverse developments for the SUSs and may serve as a protective factor against CG by providing new ways of thinking about one’s self and concerning the suicide event. Only a single cross-sectional study [16] has directly addressed the specific role of S-D in CG among SUSs. The study revealed that self-disclosure tendencies are related to lower levels of CG among SUSs, beyond the effect of other factors, such as social support and attachment style. The authors concluded that the capacity to share distressing experiences with others may offer SUSs the psychological means to better deal with their tragedy. However, no studies to date have examined the role of S-D over time as a protective factor against CG as well as against depression and suicide ideation among SUSs.

### 1.2. The Present Study

As presented in the introduction, interpersonal psychological factors in general and self-disclosure specifically have a recognized effect on mental health variables, particularly on psychological outcomes of bereavement. Notwithstanding extant data addressing the role of S-D in creating positive change among SUSs [28,29,30] and data relating to lower levels of CG, the degree to which S-D may impart a positive longitudinal effect on CG levels among those bereaving a significant other following suicide remains unclear. In this study, we examined the effect of the tendency to disclose personal and emotional information (S-D) on CG in an 18-month longitudinal design. To fully understand the psychological processes S-D may facilitate, we also examined the effect of S-D on depression levels, a symptom closely related to CG. To the best of our knowledge, the current study is the first effort at investigating interpersonal facilitators of CG among SUSs over time. The study results may help design and refine specific therapeutic interventions for SUSs that may diminish CG levels for this group.

Thus, three hypotheses were posited for this study:

**Hypothesis 1**: A. SUSs with higher levels of CG at T1 will be lower on S-D levels at both T1 and T2). B. SUSs with higher levels of CG at T1 will be higher on depressive symptoms and CG levels at T2.

**Hypothesis 2**: S-D levels will differentiate among CG levels for SUSs, and the differences between low and high levels of S-D will be higher for CG at T1 than at T2.

**Hypothesis 3**: Higher levels of S-D at T1 will contribute to lower CG levels at T2, above and beyond the trajectory of CG.

## 2. Method

### 2.1. Participants

One hundred eighty-nine SUSs participated at the initial measurement point (T1) in a study for which data collection was carried out in 2015–2016. Of these, 156 (82.5%) provided data at the second measurement time (T2), 18 months following T1. Among the remaining participants, 20 could not be reached, and 13 did not reply to the T2 recruitment letter. Demographic and psychological characteristics did not distinguish between the participants at T1 and T2. Thus, full data were collected for 156 participants in the final sample (age range 18–70; 132 females, 24 males). For a full description of the sample, see elsewhere [16,31].

An SUS was defined as someone who had lost a family member or a close friend to suicide [32]. Inclusion criteria were being at least 15 years of age at the time of the suicide and the ability to speak and write in either English or Hebrew (proficiency in either language was an inclusion criterion).

*Demographic information*. At T1, the sample’s mean age was 40.8 (*SD*_age_ = 14.58). Of this sample, 25% were aged 52–70, and 18% were between the ages of 15 and 25. Regarding the participants’ family status, 72 (46%) were married, and 61 (39%) were single. The participants reported their religiosity as mostly not religiously observant (115, 73%), with a minority (30, 19%) reporting to be religiously observant. Relating to socioeconomic status (SES), 36 (23%) participants reported a very low SES, 39 (25%) low SES, and a similar proportion of participants (38, 24%) reported medium and high SES. Regarding schooling, almost all participants (*n* = 155, 98.1%) reported completing at least 12 years.

*Suicide-related demographic information*. The participants reported various levels of relationship to the deceased: 29 parents (18.6%), 26 children (16.7%), 43 siblings (27.6%), 16 spouses (10.3%), 13 (8.4%) other family relatives, and 29 (18.6%) best friends. All reported being devastated by the suicide, ranging between *extremely devastated* (48, 30.8%) to *devastated* (21, 13.4%).

Time since the suicide varied among the participants (*M*_months_ = 80), with a range of 6 to 200 months). Twenty-eight participants (18.6%) had lost their significant other within 24 months prior to T1, 39 (25 %) within 24–48 months, 45 (29 %) within 48–72 months, and the remainder (44, 28%) six years or more prior to T1. At the time of the suicide, participants’ mean age was 31.1 (*SD*_age_ = 15.3), ranging from age 16 to 62.

### 2.2. Measures

*Self-Disclosure*. The Distress Disclosure Index (DDI; [33] was used to measure the tendency to disclose of personally-distressing information (e.g., “I usually don’t share issues that bother me” [reverse scored]; “I try to find people to talk to about my problems”). The 12-item DDI is presented on a 5-point Likert-type scale, ranging from 1 (*strongly disagree*) to 5 (*strongly agree*). The DDI’s confirmatory factor analysis suggested a single construct, yielding high reliability and validity coefficients [33]. For the DDI, higher scores were indicative of higher disclosure levels. Cronbach’s alpha for the current sample was 0.90.

*Complicated Grief*. Complicated grief levels were measured by the Inventory of Complicated Grief-Revised (ICG-R; [34], a short version of the ICG [35]. The ICG-R short-form [36] included only items applied to the CG consensus criteria and obtained correct classifications of 93% and excellent interrater agreement (k = 1.0) [37,38]. The items, presented on a 5-point Likert-type scale, tapped symptoms of separation distress and traumatic distress (e.g., inquiring about the frequency of intensity of loneliness since the loss of the deceased, intensity of yearning for the deceased). The total summed score was obtained (ranging between 17–85), with higher scores indicating higher levels of CG severity. A score above the cut-off point of 35 indicates the presence of complicated grief [35,39]. This cut-off point was used to distinguish between pathological CG SUSs (>35) and non-pathological CG (< 35). Cronbach’s alpha for the current sample was 0.95.

*Depression.* Depression levels were assessed by the Patient Health Questionnaire Depression Scale (PHQ-9; [40], a 9-item depression screen used to assess depressive symptoms. Participants were asked, “Over the last two weeks, how often have you been disturbed by any of the following problems?” They then rate the frequency of each of the symptoms on the following four anchors: 0 (*not at all*), 1 (*several days*), 2 (*more than half of the days*), to 3 (*nearly every day*). The mean of scores for the scale was used for this study. The PHQ-9 is known to be associated with increased medical visitation, physical disability, risk of psychiatric comorbidity, and overall syndromic severity [40]. The reliability of the PHQ-9 for the current sample was Cronbach’s α = 0.92.

Demographic and personal characteristics concerning the suicide loss were collected for all participants, including the participant’s relatedness to the person who died by suicide (e.g., parent, spouse, child), the time since the suicide, the survivor’s and the deceased’s ages at the time of suicide, and the extent to which the suicide was expected or unexpected.

### 2.3. Procedure

The study was approved by the Ruppin Academic Center’s ethics committee. Most participants were recruited primarily through a nonprofit organization—*Path to Life*—which is the official agency for suicide survivors in Israel. Additional participants were recruited through the Israeli suicide survivors’ online forum (“People whose loved ones died by suicide”), the SUSs’ social media group in Israel, and notices on social media sites and newspapers. All interested parties were informed of the risks and compensation procedures prior to participation. They were promised at the outset that questionnaire data would be completely anonymous, with no personal identifying information collected. Consenting participants completed the questionnaire online (using Qualtrics online survey software).

### 2.4. Data Analysis

We carried out three types of analyses. First, a multivariate analysis of variance (MANOVA) was conducted to assess the differences between pathological and non-pathological CG on S-D at T1, S-D at T2, and depression levels at T2. Secondly, repeated measures MANOVA were conducted to assess the prediction of CG at T2 from CG at T1 as a function of high and low S-D levels at T1. Finally, structural equation modeling (SEM) using AMOS was applied to assess the trajectories of CG over time and the contribution of S-D to CG at T2, beyond the CG trajectory. To obtain a full representation of the model, a combined rule for model fitness with the following accepted values was defined: a non-significant Chi-square test (*χ*^2^), anormed fit index (NFI) > 0.95 [41], a root mean square error of approximation (RMSEA) < 0.06 [42], and a standardized root-mean-square residual (RMR) < 0.08 [43]. The Statistical Package for the Social Sciences (SPSS, version 25, SPSS Inc., Chicago, IL, USA) was used for most of the analyses, and AMOS, Version 25 for Windows, was used for the SEM analysis.

## 3. Results

### 3.1. Differences between Pathological and Non-Pathological CG

**Figure 1:**
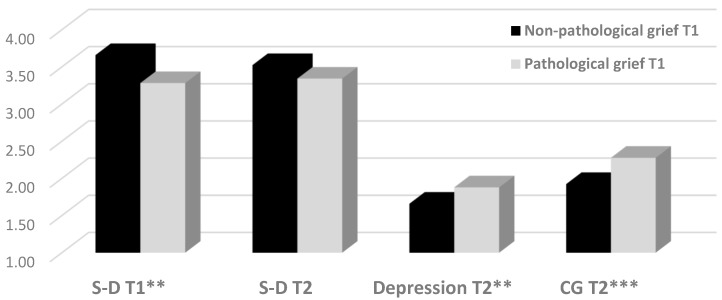
Differences between pathological and non-pathological CG in the study variables (*N* = 156). S-D = self-disclosure; CG = complicated grief; ** *p* < 0.01; *** *p* < 0.001.

We used a 2 X 3 MANOVA analysis with CG at T1 (non-pathological *n* = 68 vs. pathological *n* = 70) as the independent variable and S-D at T1, S-D at T2, CG at T2, and depression at T2 as the dependent variables. Time since suicide was used as a covariate. The overall model was statistically significant, *F*_(4,132)_ = 5.60, *p* < 0.001, η^2^ = 0.15. The ANOVA analyses revealed a significant group effect of all the dependent variables, aside from S-D at T2. As shown in Figure 1, the SUSs with pathological CG at T1 were significantly lower in S-D at T1, *F*_(1,135)_ = 7.99, *p* = 0.005, η^2^ = 0.06, and significantly higher in both CG, *F*_(1,135)_ = 17.25, *p <* 0.001, η^2^ = 0.11), and depression, *F*_(1,135)_ = 7.03, *p* = 0.009, η^2^ = 0.05) at T2.

### 3.2. Differences in CG as a Function of S-D Level

**Figure 2:**
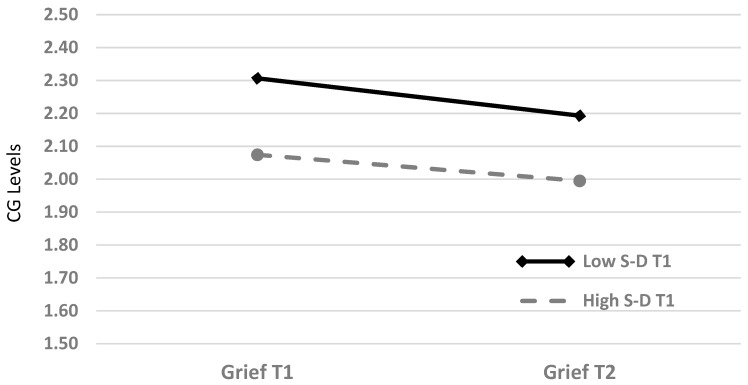
Differences between CG-T1 and CG-T2 as moderated by high and low S-D T1. S-D = self-disclosure; CG = complicated grief.

To examine the difference between CG at T1 and at T2 in relation to S-D levels, we divided the sample into two groups, by S-D median (=3): low S-D at T1 level (*n* = 81) and high S-D at T1 level (*n* = 75). We conducted a 2 X 2 MANOVA repeated measures analysis with CG at T1 and at T2 as the dependent variables and S-D groups (low and high) as the independent variable with time since suicide and depression as covariates. Two significant main effects emerged, one for time (T1 to T2), *F*_(1,152)_ = 4.07, *p* = 0.04, and one for the S-D groups, *F*_(1,152)_ = 7.41, *p* = 0.007). The interaction between group and time was not statistically significant. As can be seen in Figure 2, CG lessened over time for both groups and SUSs, with low S-D levels suffered more from CG at both T1 and T2.

### 3.3. The Contribution of S-D to the Trajectory of CG Among SUSs

**Figure 3:**
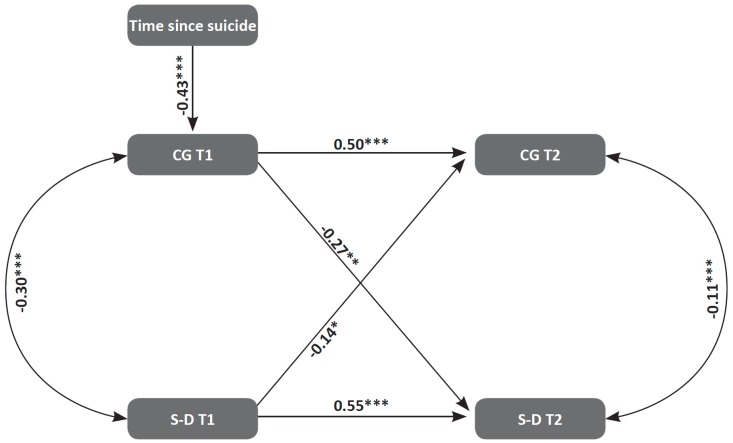
A serial mediational integrated model for CF T2 by S-D and time since suicide among suicide survivors. The rectangles indicate measured variables and the small circles reflect residuals (e). The numbers above or near endogenous variables represent the amount of variance explained (R²). The unidirectional arrows depict hypothesized directional links. Standardized maximum likelihood parameters were used. (*N* = 156). CG = complicated grief. S-D = self-disclosure. Note: * *p* < 0.05; *** *p* < 0.001.

To test the third hypothesis, which suggested that higher levels of S-D at T1 would contribute to CG levels at T2, above and beyond the natural trajectory of CG, a structural equation model (SEM) was designed, following Hayes’s [44] recommendation. The model contains the two CG and two S-D variables (T1 and T2), with the use of time since loss as a covariate. Overall, the chi-square goodness-of-fit index and the combination of values of the model presented an excellent fit with the data, χ²(3) = 0.309, *p* = 0.96); NFI = 0.99; RMSEA = 0.00; standardized RMR = 0.009. The statistically significant path coefficients are provided as standardized estimates in Figure 3. As can be seen, the analysis revealed CG’s stability over time, as well as the stability of S-D. Time elapsed since the suicide predicted (negatively) only CG at T1, meaning that CG diminished with time. Importantly, S-D at T1 negatively predicted CG at T2 above the natural trajectory of CG, meaning that higher S-D at T1 is predictive of lower CG at T2, beyond the natural reduction of CG over time. Moreover, CG at T1 was found to contribute negatively to S-D at T2 beyond the trajectory of S-D.

## 4. Discussion

Suicide is a devastating and painful phenomenon which dramatically harms those who are left behind––the suicide survivors (SUSs). According to recent studies, SUSs seem to be characterized by several psychologically adverse outcomes, among them, shame, guilt, stigma, and loneliness [7,15,45]. Furthermore, SUSs comprise an at-risk population for CG, which seems to be at the center of the adverse consequences they experience [7,30]. CG impacts many aspects in the life of SUSs, including increasing the risk for other psychopathologies, such as depression and suicide ideation. In this study, we sought to examine the trajectory of CG among SUS over time and to understand the plausible role of S-D in this trajectory. To the best of our knowledge, the longitudinal path of CG among SUSs and, more specifically, the ability of S-D to ease CG levels over time, has yet to be examined.

Our findings suggest that S-D may have an important role in dealing with grief after a loved one’s suicide. The results show that the course of CG was relatively stable over an 18-month period, and SUSs with high levels of CG at T1 remained higher at T2, compared with SUSs having low levels of S-D. Moreover, we found that SUSs with pathological CG levels at T1 reported higher levels of depression at T2 as well as lower levels of S-D at both measurement times. These results are in line with several studies that highlight the deleterious effect of post-suicide bereavement on CG and depression [46]. More central to our present study, we found that S-D can serve as a protective factor against CG over time. Overall, we found that CG levels diminished over time, but importantly, SUSs having high S-D levels at T1 presented significantly lower levels of CG at both measurement times. Upon examining the CG trajectory in the SEM model, we found that S-D at T1 contributed significantly (and negatively) to CG at T1, above and beyond the natural course of decline in CG. In other words, higher S-D levels at baseline facilitated lower levels of CG at T1 and a more profound course of healing from CG. Several studies have shown that even confiding in one significant other may facilitate adjustment and recovery [47] and might even help alleviate suicidal thoughts and behavior [48,49]. A recent report by Feigelman, Cerel, and Sanford [50] demonstrated that high levels of S-D associated with the death of a closely related person (suicide and accidental deaths) were significantly associated with a lessening of mental health issues and even diminished grief.

### 4.1. The Role of S-D as Protective Factor Against CG among SUSs

The findings emphasize the importance of S-D in CG processes among SUSs. In general, interpersonal factors have been well documented as having a protective and positive influence for those experiencing different kinds of traumas and distress [51,52]. While several studies have documented the constructive influence of S-D and interpersonal interaction in general on posttraumatic growth [53,54], much less information is available on the facilitation of S-D on CG among SUSs.

What can explain the findings regarding S-D influence? In other words, what may account for the fact that low levels of S-D have a negative effect on the potential of CG among SUSs? Observing the current findings, some possible explanations warrant mentioning. Firstly, self-disclosure and social interaction may have a meaningful role in organizing the intrapsychic response to bereavement and distress. Thus, individuals with lower tendencies to disclose personal emotions and thoughts are unlikely to accomplish a constructive mental perspective regarding their loss of loved ones [55]. This is especially significant for SUSs because they commonly deal with unsubstantiated and devastating feelings, such as shame, blame, and guilt [15]. 

Talking with family members, friends, and with other SUSs can help survivors consider different understandings regarding their mental and interpersonal state as offered by significant others and, as a result, may obtain new inner answers to the common conundrum of ‘why’ as it relates to the reasons behind the suicide event. These kinds of social communications may facilitate survivors’ recovery by dealing more effectively with the negative aspects of the suicide loss [56]. From a different angle, sharing personal and emotional information about what has occurred may help SUSs to craft new narratives even concerning the circumstances of the suicide event. Pennebaker and Keough [57] noted that “[S-D] helps people to gain meaning about their experiences, reframe these experiences as non-threatening, and assimilate them into the self…” (pp. 109–110). Among SUSs, such a change in the meaning of the suicide event and the death of the loved one can take place, in part, through talking about their overwhelming feelings and ideations. When the ability to communicate these feelings is impeded, the prospects of overcoming CG and other negative consequences relating to suicide decline considerably. 

Moreover, talking and sharing private feelings and thoughts have already been noted as important to the grief work needed in order to deal with the loss [58]. As Lepore et al. [59] suggested, social sharing of grief may be a strategy to facilitate emotional desensitization: “by talking with supportive and empathic others, trauma survivors may be able to contemplate and tolerate aversive trauma-related thoughts for a longer period of time than they would on their own” [59] (p. 271). Several studies have highlighted the phenomenon that sharing thoughts and feelings can, in some cases, even have a healing effect in at-risk populations [54] and can have a critical role in facilitating constructive intra-psychic processes [60], which, in turn, can diminish the intensity of mourning and the CG levels among survivors. Furthermore, disclosing harsh feelings may produce a “cathartic effect” [61], sometimes described as “getting it off my chest,” which has been acknowledged as one of the benefits of S-D [62]. In conclusion, whereas vigorous debate in the literature regarding the impact of disclosure on the bereaved individuals remaining behind [63], our findings highlight the import role of interpersonal behaviors, such as talking and sharing, regarding the risk of CG for SUSs.

Secondly, for individuals with only a limited level of S-D tendencies, these low S-D inclinations may buffer their engagement with their social environment, possibly leading to solitude and loneliness [64]. In this condition of isolation, it seems obvious that SUSs may encounter difficulties in initiating steps toward generating psychological changes and modifications. Following that, there is danger that CG levels would exacerbate. 

Relating to healing processes, it was found that SUSs with low levels of S-D achieved significantly lower levels of PTG in comparison with SUSs with high S-D levels and with other bereaved individuals (after traumatic or predicted loss) [65]. Furthermore, high S-D levels may help survivors adjust the nature of their interpersonal circumstances after the suicide loss. In their sharing personal and distressing information with significant others, emotional aspects of the trauma are may be revealed, leading to higher levels of intimacy, togetherness experiences, and a perceived social support [66], thus dissolving the *walls* of stigma [67] and distance. Disclosing and sharing the inner self in the context of a social support may be integrated into a sense of belongingness, which has already been highlighted as a protective factor to psychopathology and one of the fundamental human psychological needs [68]. 

Some scholars have asserted that belongingness contributes uniquely and positively to effective coping with stressors [69] and serves as a protective factor in related to distress, psychopathology and even suicidal behavior [70]. In several studies, we were able to demonstrate the protective effect of self-disclosure among SUSs in terms of increasing belongingness and perceived social support experiences, which, in turn, diminish CG and, moreover, facilitate PTG and recovery [28,30]. In these cross-sectional studies, high S-D SUSs were largely more protected against CG and showed elevated levels of PTG, compared with low S-D SUSs.

### 4.2. Limitations

The current study has some limitations. First, this study relied on self-report measures. This is common in trauma studies; however, as in other studies of this magnitude, this methodology may risk reporting bias. Future studies should seek to gather data from other sources and make use of more objective measures. Another related limitation concerns the nature of the questionnaires used in our study. Whereas our study focused on the loss of an immediate family member to suicide, measures tailored to assess SUSs are currently unavailable. Thus, similar findings might be obtained when studying other types of trauma. A related limitation is that there was no control group containing death not due to suicide. Future research should make a rigorous effort to identify factors specific to SUSs, thereby facilitating the identification of more specific therapeutic interventions for this group. We also recommend that future studies measure psychiatric histories and current diagnoses of the sample in order to gain a full picture of the emotional characteristics of the participants.

With regard to self-disclosure measurement, an important limitation is related to the fact that the DDI questionnaire that was used in our study taps the ‘tendencies toward self-disclosure’ and not the ‘actual sharing one’s personal feelings’. In other words, the results indicate the willingness of sharing rather than whether or not participants actually have disclosed their feelings about their suicide loss, how many times they did it and to whom they disclosed. Future studies are encouraged to build on this data and focus on the actual sharing of information about suicide loss and distress, and to better examine whether or not the SUSs have spoken to someone about their suicide loss, as well as to understand the specific characteristics of this sharing such as topics, length and whether or not that person was a professional/non-professional, family member/friend/stranger and other related features. 

A major limitation of this study is the use of a snowball sampling technique to increase the sample size. This limitation, together with a disproportionality female sample, may additionally limit the generalizability of the data. Future studies aimed at understanding CG among survivors should seek to recruit a more representative sample of SUSs from large community samples. Future studies should also make an effort to sample SUSs that are in similar stages of grief (maybe using time that elapsed since the suicide as an indicator of this). This may reinforce our conclusions regarding the trajectories of S-D and CG. As women are typically characterized by higher levels of S-D, gender should be taken into account in future research on this topic. Furthermore, as S-D is also closely connected to personality traits, such as introversion, introverted or shy individuals may have greater challenges in disclosing which may in turn have implications for higher likelihoods of experiencing complicated grief and other deleterious outcomes. Simultaneously assessing whether higher levels of self-disclosure is a function of a given personality trait would be helpful in teasing out direct and indirect impacts on complicated grief and may have implications for therapeutic modalities utilized to aid in self-disclosure. The use of an Israeli sample with specific cultural scripts in terms of grief and sharing of experiences may limit our ability to generalize the findings to other cultures. Lastly, while this study represents a novel effort to understand the course of CG over time among SUSs, the time gap between measurements was rather brief (eighteen months). Future studies should extend the data collection to longer periods and incorporate several measurement times to attain a fuller picture of CG’s trajectories and its moderators.

## 5. Conclusions

The present study emphasized the importance role of the tendency toward self-disclosure (S-D) on complicated grief (CG) among suicide survivors (SUSs). The findings highlight that while CG was highly prevalent among SUSs, S-D has a beneficial effect which can serve as a protective factor against CG among this group. The clinical experience of the authors indicates that SUSs often have difficulties in talking and sharing their personal tragedy with others. In some cases, SUSs are inclined to dismiss relationships with significant others, which, in turn, leads to loneliness and detachment. Consequently, lack of S-D (and termination of relationships) was found to facilitate CG, which may lead to other psychological deteriorations, such as sleep problems, depression, and suicide ideation. However, those able to share with others are likely to feel a sense of support which, in turn, can better protect them against CG and other deleterious effects of bereavement.

Therapy techniques, such as interpersonal therapy (IPT) [71], designed to improve interpersonal skills in general and S-D abilities specifically may be effective in individual or group psychotherapy for SUSs. This approach, which represents the notion of “let’s talk about it”, may assist survivors to overcome the personal tragedy of losing a family member to suicide and to facilitate their recovery from their struggle with these shattering circumstances.
